# Amongst Women Stratified to Receive Endocrine Therapy on the Basis of Their Tumor Estrogen and Progesterone Receptor Levels, Those with Higher Tumor Progesterone Receptor Levels Had a Better Outcome Than Those with Lower Levels of Tumor Progesterone Receptor

**DOI:** 10.3390/cancers13040905

**Published:** 2021-02-21

**Authors:** Tai-Han Lin, Hong-Wei Gao, Guo-Shiou Liao, Jyh-Cherng Yu, Ming-Shen Dai, Jar-Yi Ho, Cheng-Ping Yu

**Affiliations:** 1 Department of Pathology and Graduate Institute of Pathology and Parasitology, Tri-Service General Hospital, National Defense Medical Center, No. 161, Section 6, Minquan E. Road, Neihu District, Taipei 114, Taiwan; 402010258@mail.ndmctsgh.edu.tw (T.-H.L.); doc31796@mail.ndmctsgh.edu.tw (H.-W.G.); 2 Department of Surgery, Tri-Service General Hospital, National Defense Medical Center, Taipei 114, Taiwan; guoshiou@ndmctsgh.edu.tw (G.-S.L.); doc20106@ndmctsgh.edu.tw (J.-C.Y.); 3 Department of Medicine, Tri-Service General Hospital, National Defense Medical Center, Taipei 114, Taiwan; wen17140@mail.ndmctsgh.edu.tw; 4 Graduate Institute of Life Sciences, National Defense Medical Center, No. 161, Section 6, Minquan E. Road, Neihu District, Taipei 114, Taiwan

**Keywords:** breast cancer, hormone therapy, progesterone receptor, estrogen receptor

## Abstract

**Simple Summary:**

Breast cancer is the most commonly diagnosed cancer and the leading cause of cancer death of women worldwide. Several cut-points for estrogen receptor (ER) and progesterone receptor (PgR) have been proposed as predictive effects of hormone therapy; while the cut-off values were inconsistent. The aim of our retrospective study was to propose better prognostic cut-off levels for ER and PgR, and their effects on breast cancer-specific survival (BCSS) and disease-free survival (DFS) over 5 and 10 years were evaluated in 1807 eligible patients. Subgroups were generated based on ER and PgR expression percentage and scoring from the Allred scoring system (Allred scores). After comparing the hazard ratios (event rates in each group to reference group) of BCSS and DFS using multivariate analyses, our results suggested that patients with PgR expression ≤50% or Allred score ≤5 revealed a poor prognosis and should be paid more attention during follow-up.

**Abstract:**

Background: To realize the association between stratified expression levels of ER and PgR and long-term prognosis of breast cancer patients who received adjuvant hormone therapy, this study aimed to propose better prognostic cut-off levels for estrogen receptor (ER) and progesterone receptor (PgR). Methods: Patients who received adjuvant hormone therapy after surgical intervention were selected. The ER and PgR status and their effects on breast cancer-specific survival (BCSS) and disease-free survival (DFS) over 5 and 10 years were evaluated. Next, subgroups were generated based on ER and PgR expression percentage and Allred scores. Survival curves were constructed using the Kaplan–Meier method. Results: ER and PgR expression were significantly associated with better prognosis in 5 years, whereas only PgR expression was significantly associated during the 10-year follow-up. The optimal cut-off values for better 5-year BCSS were ER > 50%; ER Allred score > 7; PgR ≥ 1%; or PgR Allred score ≥ 3; the corresponding values for DFS were ER > 40%; ER Allred score > 6; PgR > 10%; or PgR Allred score ≥ 3. In the long-term follow-up, PgR of > 50% or Allred score of > 5 carriers revealed a better prognosis of both BCSS and DFS. Conclusion: Patients with a PgR expression > 50% or an Allred score > 5 exhibited better 10-year BCSS and DFS.

## 1. Introduction

Among females, breast cancer (BC) is the most commonly diagnosed cancer and the leading cause of cancer death worldwide [1]. BC can be classified into luminal, human epidermal growth factor receptor-2 (HER2), or triple-negative subtypes after immunohistochemical (IHC) analysis of the estrogen receptor (ER), progesterone receptor (PgR), HER2, and Ki-67. Prognostic information can be predicted based on the IHC profile. Furthermore, individualized therapies should be developed according to the subtype of BC. Generally, patients who present with the luminal subtype are subjected to hormone therapy [2]. Those with HER2 subtype exhibit relatively poorer outcomes, but they can be treated with anti-HER2 targeted therapy; likewise, triple-negative subtypes BCs demonstrate a poor prognosis and can optimally be treated via chemotherapy, immunotherapy, or antibody-drug conjugate for advanced staged patients, or targeted therapy with poly(adenosine diphosphate-ribose) polymerase inhibitors in advanced patients with germline BRCA1 or BRCA2 mutation [2,3,4,5]. Among the aforementioned biomarkers, ER is the most important considering its crucial roles in carcinogenesis and hormone therapy [2].

According to the American Society of Clinical Oncology (ASCO)/College of American Pathologists guidelines (CAP), ER and PgR assays are considered positive if at least 1% of the tumor cell nuclei in the sample are immunoreactive [6]. About 65–80% of BCs express ER, are usually better differentiated, respond better to hormonal therapy, and demonstrate a favorable prognosis when compared to ER-negative tumors [7]. Furthermore, 65–75% of BCs express PgR, which is mostly co-expressed with ER. The absence of PgR expression has been associated with a poorer prognosis and worse response to hormonal therapy [7].

Despite the distinct subtypes, significant reductions in BC recurrence and mortality rates can be achieved with proper adjuvant systemic therapy. Hormone therapy is recommended for most patients with ER+ or PgR+ BC [8]. Several criteria, cut-points, or calculated formulas for ER and PgR have been positively associated with BC prognosis and the therapeutic efficacy of hormone therapy, particularly, tamoxifen [9,10,11,12]. The predictive effects of ER and/or PgR levels were mostly fractioned as >1% [9,10] or >10% [11,12] positivity in BC specimens; however, the discrete proportion of the positivity did not reflect whether the effect varies over time or is increased with the increase in the expression levels of the receptors [13]. Currently, the number of low ER-positive patients is very low (≤10%), and more evidence is needed to evaluate the treatment benefits in these patients [6]. Moreover, hormone receptor-positive patients with higher Allred score (6–8) also revealed better prognosis than those with lower Allred scores (3–5) to access the response of oophorectomy plus tamoxifen [14]. Furthermore, regarding confirmation of low PgR-positive group and whether an increase in the level of PgR expression correlates with a better prognosis, limited evidence exists [6].

The aim of this study was to investigate the quantitative effects of ER and PgR on BC prognosis after hormone therapy. Furthermore, subgroups of patients were generated based on the positive expression percentages and Allred scores [15] to analyze the differences between disease-free survival (DFS) and breast cancer-specific survival (BCSS).

## 2. Results

### 2.1. Characteristics of the Study Population

During the follow-up period, 122 patients died from causes attributable to BC and 138 patients exhibited local or distant recurrence. The median BCSS of all patients was 95 months (interquartile range, 70–125 months), with a 5- and 10-year rate of 96.2% and 90.1%, respectively. The median DFS was 92 months (interquartile range, 66–123 months), with a 5- and 10-year rate of 92.0%, and 87.0%, respectively. Patients who received breast-conserving surgery (BCS) had longer BCSS and DFS than patients who received modified radical mastectomy (MRM). Patients with smaller tumor size had longer BCSS and DFS than patients with larger tumor size. Patients without lymph node involvement had longer BCSS and DFS than patients with lymph node involvement. Patients with ductal carcinoma had longer BCSS and DFS than patients with lobular carcinoma or mixed ductal-lobular carcinomas. Patients with histological grade I tumors have longer BCSS and DFS than patients with histological grade II and III tumors. Patients with positive ER/PgR had longer BCSS and DFS than patients without expression. Patients with strong ER/PgR intensity had longer BCSS and DFS than patients with weak intensity. Patients who received tamoxifen had longer BCSS and DFS than patients who received other hormone therapies. Patients who received chemotherapy had shorter BCSS and DFS than patients without chemotherapy.

The clinicopathological characteristics of the recruited patients were classified according to the targeted events (BCSS and DFS) within 5 years (Table 1). With regard to the BCSS status, the median ages of the patients who died of (range, 29–89 years) and survived from (range, 23–90 years) BC were 53 and 51, respectively. To evaluate the association between each prognostic risk factors list in Table 1 and BCSS of the recruited cohort, Pearson’s chi-square test was used to analyze different distribution of each factor. Significant differences in surgical type (*p* < 0.001), tumor size (*p* < 0.001), lymph node status (*p* < 0.001), histologic grade (*p* = 0.011), ER intensity (*p* = 0.029), PgR status (*p* = 0.001), PgR intensity (*p* < 0.001), hormone therapy type (*p* < 0.001), and chemotherapy (*p* < 0.001) were observed between the two groups.

With regard to the DFS status, the median ages were 53 and 51 among those with (range, 29–89 years) and without (range, 23–90 years) BC recurrence, respectively. Pearson’s chi-square test was also used to analyze different distribution of each factor among DFS status of the recruited cohort. Significant differences in surgical type (*p* < 0.001), tumor size (*p* < 0.001), lymph node status (*p* < 0.001), histologic grade (*p* = 0.001), ER status (*p* = 0.014), ER intensity (*p* = 0.029), PgR status (*p* = 0.002), PgR intensity (*p* = 0.001), hormone therapy type (*p* < 0.001), and chemotherapy (*p* < 0.001) were observed among the two groups.

### 2.2. Treatment Data

Among all patients who were recruited in this study, 1122 (62.1%) were treated with MRM and 685 (37.9%) were treated with BCS. Furthermore, five years of hormone therapy was arranged for recruited patients, 1146 patients (63.4%) were treated with tamoxifen and 195 (10.8%) were treated with aromatase inhibitors (anastrozole, exemestane, or letrozole); 466 patients (25.8%) were initially treated with tamoxifen and were changed to aromatase inhibitors two or three years later. In addition, 1015 patients (56.2%) received chemotherapy and 877 (48.5%) received radiotherapy.

### 2.3. Cox Regression Analyses of Variables That Affected the Clinical Endpoints after Five Years and Ten Years

In the BCSS status analysis, MRM, larger tumor size, lymph node involvement, high histological grade, hormone therapy other than tamoxifen, and chemotherapy were significantly associated with an increased risk of BC-specific mortality in the univariate analysis (Table 2); alternatively, ER (HR, 0.56; 95% CI 0.32–0.98; *p* = 0.042) and PgR (HR, 0.347; 95% CI, 0.196–0.616; *p* < 0.001) expression significantly reduced the risk. No significant prognostic effect of adjuvant radiotherapy was observed. In a multivariate model, which included the standard clinical and pathological factors significant in the univariate analysis, MRM, larger tumor size, and lymph node involvement remained as the independent prognostic factors for BCSS events. Moreover, ER (HR, 0.482; 95% CI, 0.260–0.895; *p* = 0.021) and PgR (HR, 0.298; 95% CI, 0.162–0.548; *p* < 0.001) positivity were the independent protective factors for BCSS events. The aforementioned variables, including MRM, larger tumor size, lymph node involvement, ER and PgR positivity, and hormone therapy (except for tamoxifen), acted as independent prognostic factors for DFS events in the 5-year follow-up.

Ten-year events were assessed in 717 patients diagnosed with BC before 2010. With regard to the BCSS status, MRM, larger tumor size, lymph node involvement, lobular subtype, mixed ductal and lobular subtype, high histological grade, hormone therapy other than tamoxifen, and chemotherapy were significantly associated with an increased risk of BC-specific mortality in the univariate analysis (Table 3); alternatively, PgR (HR, 0.484; 95% CI, 0.263–0·888; *p* = 0.019) expression significantly reduced the risk. No significant prognostic effect of ER status, or adjuvant radiotherapy was noted. In the multivariate model, which included the standard clinical and pathological factors significant in the univariate analysis, MRM, larger tumor size, lymph node involvement, and treated with tamoxifen and aromatase inhibitors continued to act as independent prognostic factors for BCSS events, whereas PgR positivity (HR, 0.374; 95% CI, 0.234–0.597; *p* < 0.001) acted as an independent protective factor for BCSS events. The aforementioned variables, including MRM, larger tumor size, lymph node involvement, PgR positivity, and hormone therapy (except for tamoxifen), acted as independent prognostic factors for DFS events in the 10-year follow-up.

In summary, ER and PgR expression were significantly associated with a better prognosis in terms of the BCSS and DFS within five years after adjusting the other clinicopathological covariates in the multivariate analyses; however, only PgR expression was significantly associated with a better long-term prognosis in terms of BCSS and DFS over a period of 10 years after adjusting the other clinicopathological covariates in the multivariate analyses.

### 2.4. Optimizing the Cut-Off Value for the IHC Profiles of ER and PgR

**Figure 1:**
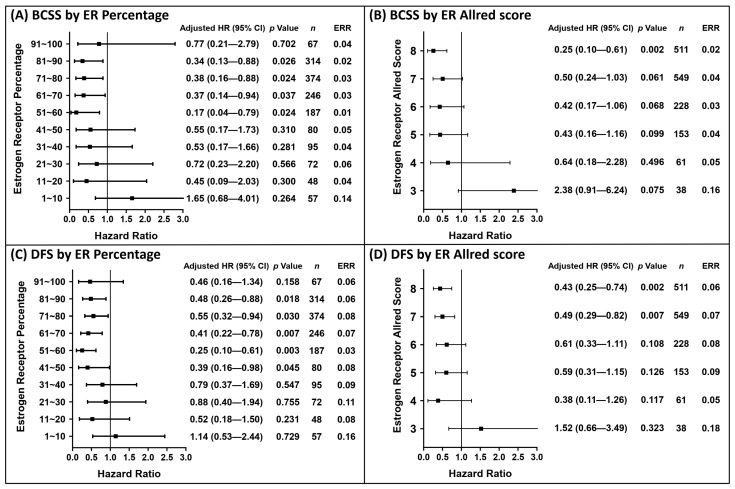
Distribution of hazard ratio based on the ER status within 5 years of follow-up. The hazard ratios were calculated after adjusting significant covariates under univariate analysis in Table 2. A total of 267 patients with negative ER expression but positive PgR expression were defined as the reference group; 16 patients died from BCSS events and 32 exhibited DFS events during the period in the reference group. Hazard ratios for BCSS events by subgroups of ER percentage (**A**) and Allred score (**B**). Hazard ratios for DFS events by subgroups of ER percentage (**C**) and Allred score (**D**).

**Figure 2:**
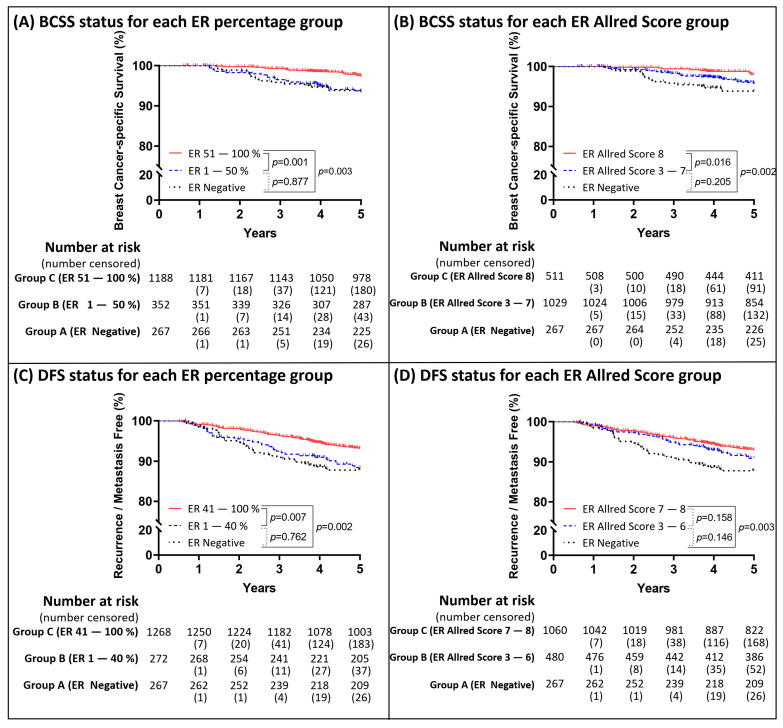
Kaplan–Meier curves within 5 years based on the ER subgroup status. The patients were divided into three groups based on the result in Figure 1. Group A as ER negative expression but positive PgR expression; Group B as low ER expression; Group C as high ER expression. Kaplan–Meier curves for BCSS were depicted based on the ER expression percentage (**A**) and Allred score (**B**). Kaplan–Meier curves for DFS on the ER expression percentage (**C**) and Allred score (**D**).

The distribution of the hazard ratio (HR) in each subgroup generated from the ER IHC profile after adjusting the other significant covariates during the 5-year follow-up are presented in Figure 1 with event rate ratios (ERR). The risk of BCSS events was significantly reduced if the ER percentage was >50% (Figure 1A) or Allred score was >7 (Figure 1B); the risk of DFS events as significantly reduced if the ER percentage was >40% (Figure 1C) or Allred score was >6 (Figure 1D). The numbers mentioned in Figure 1 were considered as cut-off values. Kaplan–Meier survival curves were plotted in Figure 2 based on the ER cut-off values obtained in Figure 1. Despite ER not being a significant variable in Table 3, the analytical results of 10-year follow-up was shown in Supplementary Figure S1. ER was not included in analysis of 10-year event due to no cut-off value being identified by difference in HRs.

**Figure 3:**
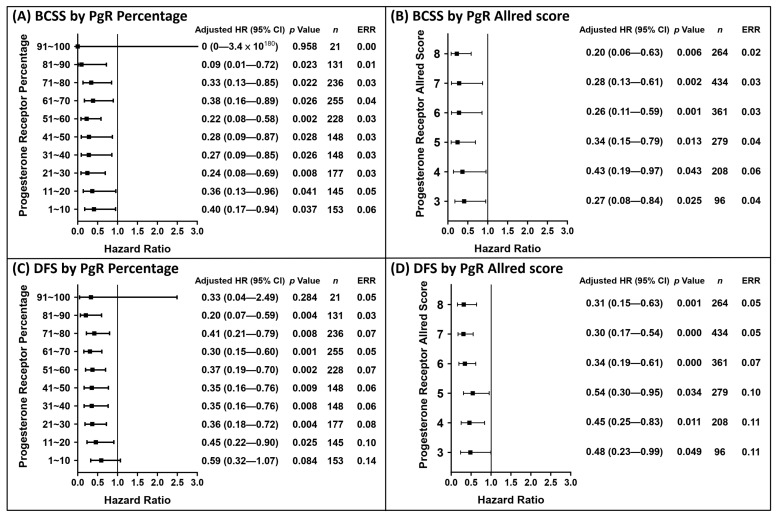
Distribution of hazard ratio based on the PgR status within 5 years of follow-up. The hazard ratios were calculated after adjusting significant covariates under univariate analysis in Table 2. A total of 165 patients with negative PgR expression but positive ER expression were defined as the reference group; 15 patients died from BCSS events and 24 exhibited DFS events during the period in the reference group. Hazard ratios for BCSS events by subgroups of PgR percentage (**A**) and Allred score (**B**). Hazard ratios for DFS events by subgroups of PgR percentage (**C**) and Allred score (**D**).

**Figure 4:**
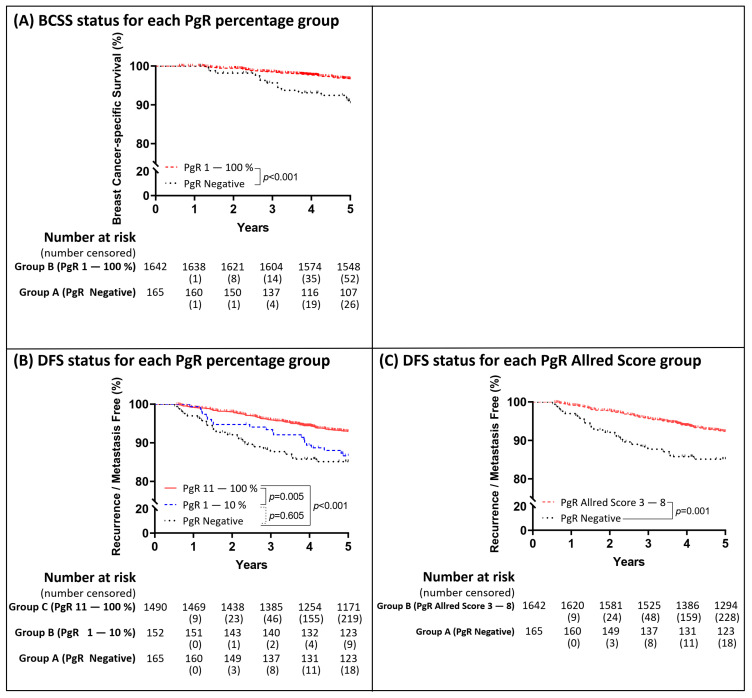
Kaplan–Meier curves within 5 years based on the PgR group status. The patients were divided into two groups (group A as PgR negative expression; group B as PgR positive expression) based on the result in Figure 3. Kaplan–Meier curves of BCSS were depicted according to the two groups (**A**); Kaplan–Meier curves of DFS were depicted according to the two groups (**C**). The patients were divided into three groups (group A as PgR negative expression; group B as low PgR expression; group C as high PgR expression) based on the result in Figure 3. Kaplan–Meier curves for DFS were depicted based on the expression percentage (**B**).

**Figure 5:**
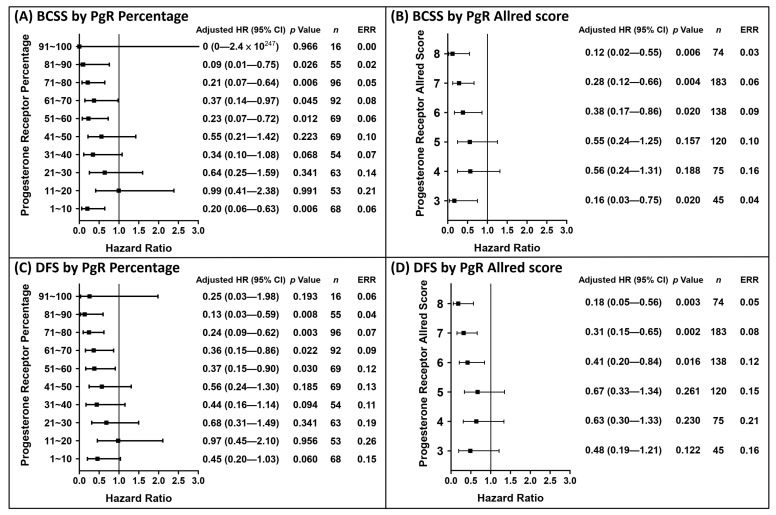
Distribution of hazard ratio based on the PgR status within 10 years of follow-up. The hazard ratios were calculated after adjusting significant covariates under univariate analysis in Table 3. A total of 82 patients with negative PgR expression but positive ER expression were defined as the reference group; 13 patients died from BCSS events and 16 exhibited DFS events during the period in the reference group. Hazard ratios for BCSS events by subgroups of PgR percentage (**A**) and Allred score (**B**). Hazard ratios for DFS events by subgroups of PgR percentage (**C**) and Allred score (**D**).

**Figure 6:**
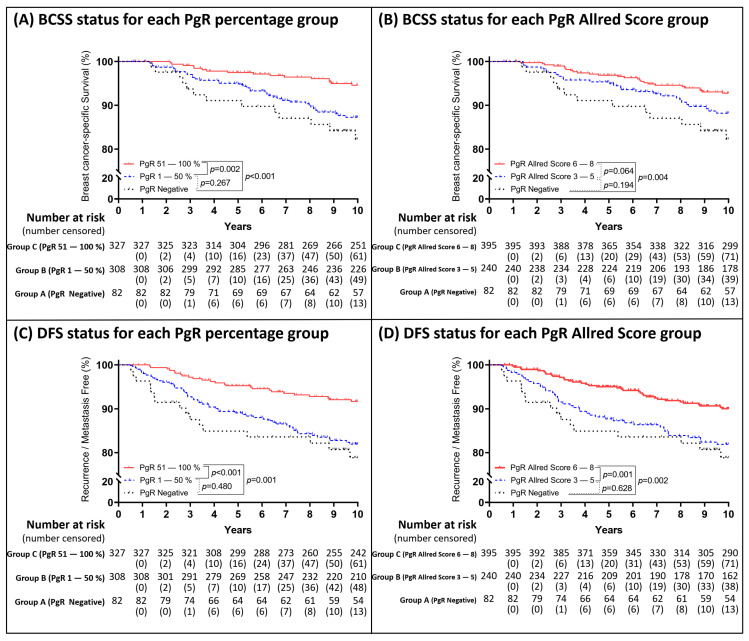
Kaplan–Meier curves within 10 years based on the PgR group status. Group A as PgR negative expression; Group B as low PgR expression; Group C as high PgR expression. The patients were divided into three groups based on the result in Figure 5. Kaplan–Meier curves for BCSS were depicted based on the expression percentage (**A**) and Allred score (**B**). Kaplan–Meier curves for DFS based on the expression percentage (**C**) and Allred score (**D**).

According to our findings, the PgR status could be used to predict the events at the 5- and 10-year clinical endpoints. The analytical results of the 5-year follow-up with regard to the PgR status are shown in Figure 3. The risk of BCSS events within 5 years was significantly reduced when the PgR percentage was ≥1% (Figure 3A) and the Allred score was ≥3 (Figure 3B), while the risk of DFS events significantly reduced when the PgR percentage was >10% (Figure 3C) and Allred score was ≥3 (Figure 3D). Based on the PgR cut-off values shown in Figure 3, the Kaplan–Meier survival curves were plotted in Figure 4. The analytical results of the 10-year follow-up are shown in Figure 5. The risks of BCSS and DFS events were significantly reduced when the PgR percentage was >50% (Figure 5A,C) and Allred score was >5 (Figure 5B,D). The Kaplan–Meier survival curves (Figure 6) were plotted based on the PgR cut-off values shown in Figure 4.

## 3. Discussion

In the present study, we retrospectively evaluated the association between prognostic risks of BCSS and DFS events and to the stratification of ER and PgR expression levels according to their percentage or Allred scores. A high PgR expression level was significantly associated with a better prognosis in both BCSS and DFS over a follow-up period of 10 years, whereas ER was only applicable for evaluating the events within a 5-year period, after adjusting the other clinicopathological covariates in the multivariate analyses.

A total of 1808 patients were selected for analysis from registry with higher estrogen receptor positivity than progesterone receptor positivity (60.6% ER-positive/PgR-positive, 7.6% ER-positive/PgR-negative, 13% ER-negative/PgR-positive, and 18.8% ER-negative/PgR-negative) after excluding ER-negative/PgR-negative and HER2 over-expressed patients in current study. Based on a serial graphical view of the HRs within a 5-year period in each subgroup stratified by percentage or Allred score compared to reference (patients with ER-positive/PgR-negative or ER-negative/PgR-positive), the cut-off values for a better BCSS were as follows: 50% ER positivity or an Allred score of 7; the corresponding values for a better DFS were as follows: 40% ER positivity or an Allred score of 6. However, we were unable to determine the cut-off value of PgR with regard to the BCSS status because a positive expression of ≥1% demonstrated a better prognosis compared to a negative expression. Alternatively, a better DFS can be hinted with cut-off value of 10% PgR positivity. For the evaluation of better prognosis over a longer period of up to 10 years, the cut-off values were 50% PgR positivity or an Allred score of 5 for both BCSS and DFS. Despite a trend of decrease in the ERR of BCSS and DFS being observed in higher expression percentage or in higher Allred score in Figure 1, Figure 3, and Figure 5, fluctuation was also noted; therefore, we could not conclude whether increase in the degree of each receptor expression correlate with better outcome linearly.

In clinical practice, IHC staining for ER and PgR status was recommended to identify BC patients with potential benefits from adjuvant hormone therapy [6]. Several studies examined the IHC status of ER and PgR to determine the optimal cut-off values that can be used to predict the therapeutic benefits of hormone therapy [9,10,11,12]. Consequently, an ER expression of 1% to 10% was classified as low ER-positive due to limited clinical evidences of the benefits from hormone therapy in these patients; hence, hormone therapy should be initiated after weighing the risks and benefits [6]. Selective estrogen receptor modulators were the most commonly prescribed medications for patients with positive ER expression due to its significant improvement in BC-related mortality and recurrence [16].

In the current study, ER+ patients accounted for 85% of the recruited cohort, and they were majorly treated with tamoxifen for 5 years. As reported in previous studies [17,18,19], fewer BCSS and DFS events were noted in the ER+ group compared to the ER− group within five years in our results. However, no statistically significant difference between the two groups was observed in the 10-year follow-up, despite the presence of a better survival trend. A sharp early peak in the HR at around 2 years in the ER-negative patients followed by a decline to values below that in the ER-positive patients after seven years might explain the loss in the prognostic role of ER during the longer follow-up period in the current study [20,21,22].

PgR expression at the molecular level could suggest the presence of a functional pathway between ER and PgR in the tumor cell after estrogen binding [23]. Paradoxical conditions, such as ER−/PgR+, can occur if some variants of ER are not detected by conventional antibodies or in the absence of an estrogen binding region due to exon deletion, but they are still capable of proceeding with the signaling pathway [24]. Furthermore, an in vitro study of BC cells with ER expression, transcriptional activity, and cell proliferation can be impeded by PgR expression [25]. Compared to those with PgR expression, patients without PgR expression were associated with a poorer outcome of BCSS and DFS [26]. ER expression between 1 and 10% was classified as low-ER positive in 2020 ASCO/CAP guidelines; however, no definition for low PgR-positive patients was stated [6]. Nordenskjöld et al. proposed that patients with a PgR of <10% exhibited no benefit after receiving tamoxifen treatment, suggesting that their clinical outcome was similar to that of PgR-negative patients [27]. Similar recurrence rates in the PgR-negative and PgR ≤10% groups were observed in the current study. Aside from IHC, other histology features including subtype and tumor grade did not correlate with shorter or longer BCSS or DFS in five or ten years after adjusting other covariates.

Furthermore, this study analyzed the statistical influence of both percentage and intensity using the Allred score. By definition, a PgR percentage of 1–10% with weak, moderate, and strong intensity was graded as 3, 4, and 5, respectively; a PgR percentage of 11–33% with weak, moderate, and strong intensity was graded 4, 5, and 6, respectively; and a PgR percentage of 34–66% with weak, moderate, and strong intensity was graded as 5, 6, and 7, respectively [14]. Combining the results of the percentage and Allred scores, we noticed that a PgR of >10–33% with strong intensity, PgR of 34–50% with moderate/strong intensity, or PgR of >50% regardless of intensity was associated with a better BCSS and DFS during the 10-year follow-up, regardless of the ER status in our cohort. Not all centers measure PgR, and it might be optional if ER was detected in some centers, and it might be suggested for detection for each center to more precisely evaluate the prognosis. Furthermore, more attention could be paid after initiating adjuvant hormone therapy in patients with low PgR positivity (PgR, ≤50% or Allred score, ≤5) since treatment resistance or failure could occur more frequently in these patients.

Several potential limitations should be considered when interpreting the findings of this study. First, the study was retrospective with a single-center design, and due to the limited sample size, the statistical power was restricted when analyzing events over a period of 10 years. Second, about 10% of the patients with ER and/or PgR positivity were excluded from the analysis due to missing data in the registry; the reduced sample sizes may have resulted in an unpredictable statistical bias. Third, subgroups numbers were more evenly distributed for PgR than ER in the current study, and our conclusion that PgR predicts prognosis better than ER relies upon the different distribution of the positivity groupings. Four, due to relatively limited data of Ki-67 staining in our registry, molecular subtypes (luminal A, luminal B, basal-like, and HER2) were not included for this analysis. However, to the best of our knowledge, the current study represents one of the largest series in the literature to date.

## 4. Materials and Methods

### 4.1. Study Population

**Figure 7:**
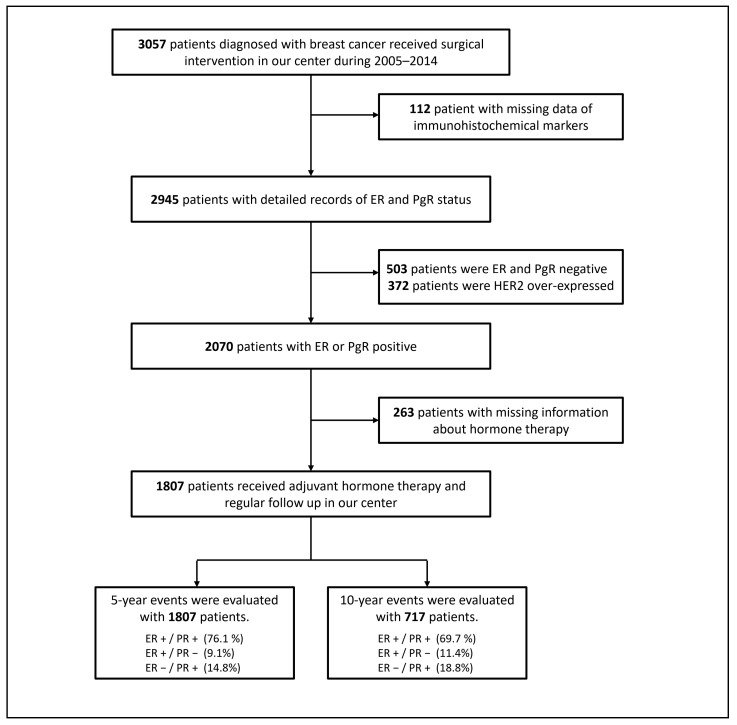
Flow diagram of the patient selection. Abbreviation: ER: estrogen receptor, PgR: progesterone receptor, HER2: human epidermal growth factor receptor-2.

All data were derived from the BC registry database at the Tri-Service General Hospital in Taiwan, from 2005–2014. The medical chart records were retrospectively reviewed under the approval of the institutional review board of the Tri-Service General Hospital NO. B202005044. The follow-up endpoint for the survivors was December 2019. The need to acquire individual informed consent was waived by the institutional review board because the data were analyzed anonymously. As shown in Figure 7, patients with a histopathologically confirmed primary diagnosis of breast carcinoma were assessed from the database. The inclusion criteria were as follows: women who received BCS or MRM; those with detailed information regarding the ER and PgR expression status; those with ER- and/or PgR-positive expression; those without HER2 over-expression; and those with records of receiving adjuvant hormone therapy. A total of 1807 eligible patients were included in the study.

### 4.2. Immunohistochemical Data Collection

All the IHC specimens for ER/PgR/HER2 were reviewed by three dedicated pathologists at the central IHC laboratory of our hospital. The subtypes were recognized according to the World Health Organization (WHO) classification [28]. ER and PgR were considered as positive if ≥1% of the tumor cell nuclei in the sample were immunoreactive, and representative IHC staining samples were as in Supplementary Figure S2 [6,29]. HER2 scoring was categorized as 0, 1+, 2+, or 3+, wherein a score of 0 or 1+ was considered negative, and a score of 3+ was considered positive; a score of 2+ was deemed equivocal and further analyzed by fluorescence in situ hybridization [30]. A case may be recognized as uninterpretable if the specimen size is inadequate for analysis.

### 4.3. Clinical and Treatment Data

The database included age at diagnosis, date of diagnosis, time to recurrence, time to death by any course, and date of final contact. A cause of death not attributable to BC was treated as censored. The tumor-related characteristics included histological subtype (ductal, lobular, mixed ductal and lobular, or others), tumor grade (low, moderate, or high), tumor size (<2 cm, 2–5 cm, or >5 cm), lymph node status (negative or positive), and treatment factors (type of surgical intervention, chemotherapy, hormone therapy, and radiotherapy). The clinical endpoints for BCSS were defined as the time to death from BC, and for DFS were defined as the time to local or distant BC recurrence, diagnosis of secondary BC, or breast cancer-related death after 5 and 10 years. The 5-year events were evaluated in all patients recruited in the study, whereas only those diagnosed with breast cancer during 2005–2009 received evaluations for the 10-year events.

### 4.4. Statistical Analysis

The participants were grouped according to the status of the BCSS and DFS at 5 and 10 years. The distribution of the IHC status and other categorical variables were compared using Pearson’s Chi-square test. A univariate and multivariate Cox regression model was used to determine the association between the clinicopathological characteristics and BCSS and DFS. Hazard ratios (HRs) for BCSS and DFS were estimated via multivariate analysis using the Cox proportional hazards model after adjustment of the statistically significant variables in the univariate analysis.

In order to understand the pattern of the treatment effect on the ER and PgR characteristics in the IHC analysis, subgroups were created based on the expression percentage and Allred score. The optimal cut-off value with a serial graphical view of the estimated HRs of the endpoint events was determined. Survival curves were constructed using the Kaplan–Meier method. All the statistical tests were performed using SPSS Statistics version 22.0 (IBM Corp., Armonk, NY, USA) with a two-sided significance level of 5%. HRs were presented with their 95% confidence intervals.

## 5. Conclusions

After comparing the HRs for BCSS and DFS in all subgroups using a multivariate analysis during a 5- and 10-year follow-up, our results suggested that higher ER or PgR expression level was associated with better 5-year BCSS and DFS among breast cancer patients received adjuvant hormone therapy. Moreover, higher PgR expression level was also associated with better 10-year BCSS and DFS in the same cohort. Although the clinical practice mainly relies on the ER expression status to initiate hormone therapy, ER lost its prognostic role in the 10-year follow-up period in the current study. Therefore, more attention should be paid to patients with PgR expression levels of <50% or an Allred score of <5 since more BCSS and DFS events occurred in these patients. Additional randomized prospective multicenter studies are warranted to confirm the findings of this study.

## Figures and Tables

**Table 1:** Clinicopathological characteristics of the breast cancer patients according to the breast cancer-specific survival (BCSS) and disease-free survival (DFS) events within five years of treatment.

Characteristic	BCSS			DFS		
	Mortality (*n* = 68)	Survival (*n* = 1739)	*p*	Recurrence (*n* = 144)	Disease-Free (*n* = 1663)	*p*
**Age**			0.802			0.599
<50	27 (39.7%)	728 (41.9%)		57 (39.6%)	698 (42%)	
≥50	41 (60.3%)	1011 (58.1%)		87 (60.4%)	965 (58%)	
**Operation type**			**<0.001**			**<0.001**
BCS	11 (16.2%)	674 (38.8%)		30 (20.8%)	655 (39.4%)	
MRM	57 (83.8%)	1065 (61.2%)		114 (79.2%)	1008 (60.6%)	
**Tumor size (cm)**			**<0.001**			**<0.001**
≤2	14 (20.6%)	1172 (67.4%)		45 (31.2%)	1141 (68.6%)	
>2, ≤5	39 (57.4%)	513 (29.5%)		77 (53.5%)	475 (28.6%)	
>5	15 (22.1%)	54 (3.1%)		22 (15.3%)	47 (2.8%)	
**Lymph node**			**<0.001**			**<0.001**
Negative	21 (30.9%)	1272 (73.1%)		62 (43.1%)	1231 (74%)	
Positive	47 (69.1%)	467 (26.9%)		82 (56.9%)	432 (26%)	
**Histological subtype**			0.337			0.058
Ductal	52 (76.5%)	1432 (82.3%)		114 (79.2%)	1370 (82.4%)	
Lobular	5 (7.4%)	109 (6.3%)		10 (6.9%)	104 (6.3%)	
Mixed (ductal and lobular)	9 (13.2%)	130 (7.5%)		18 (12.5%)	121 (7.3%)	
Others	2 (2.9%)	68 (3.9%)		2 (1.4%)	68 (4.1%)	
**Histologic grade**			**0.002**			**0.001**
1	3 (4.4%)	270 (15.6%)		8 (5.6%)	265 (16%)	
2	18 (26.5%)	622 (35.9%)		47 (32.6%)	593 (35.8%)	
3	47 (69.1%)	842 (48.6%)		89 (61.8%)	800 (48.3%)	
Unknown (*n* = 5)				-	-	
**ER**			0.052			**0.014**
Negative	16 (23.5%)	251 (14.4%)		32 (22.2%)	235 (14.1%)	
≥1%	52 (76.5%)	1488 (85.6%)		112 (77.8%)	1428 (85.9%)	
**ER intensity**			**0.029**			**0.029**
0	16 (23.5%)	251 (14.4%)		32 (22.2%)	235 (14.1%)	
1	11 (16.2%)	195 (11.2%)		18 (12.5%)	188 (11.3%)	
2	27 (39.7%)	683 (39.3%)		56 (38.9%)	654 (39.3%)	
3	14 (20.6%)	610 (35.1%)		38 (26.4%)	586 (35.2%)	
**PgR**			**0.001**			**0.002**
Negative	15 (22.1%)	150 (8.6%)		24 (16.7%)	141 (8.5%)	
≥1%	53 (77.9%)	1589 (91.4%)		120 (83.3%)	1522 (91.5%)	
**PgR intensity**			**<0.001**			**0.001**
0	15 (22.1%)	150 (8.6%)		24 (16.7%)	141 (8.5%)	
1	21 (30.9%)	454 (26.1%)		45 (31.2%)	430 (25.9%)	
2	24 (35.3%)	700 (40.3%)		52 (36.1%)	672 (40.4%)	
3	8 (11.8%)	435 (25%)		23 (16%)	420 (25.3%)	
**Hormone therapy**			**<0.001**			**<0.001**
Anti-estrogens	28 (41.2%)	1118 (64.3%)		47 (32.6%)	1099 (66.1%)	
Enzyme-inhibitors	16 (23.5%)	179 (10.3%)		28 (19.4%)	167 (10%)	
Both	24 (35.3%)	442 (25.4%)		69 (47.9%)	397 (23.9%)	
**Chemotherapy**			**<0.001**			**<0.001**
No	12 (17.6%)	780 (44.9%)		33 (22.9%)	759 (45.6%)	
Yes	56 (82.4%)	959 (55.1%)		111 (77.1%)	904 (54.4%)	
**Radiotherapy**			0.062			0.488
No	27 (39.7%)	903 (51.9%)		70 (48.6%)	860 (51.7%)	
Yes	41 (60.3%)	836 (48.1%)		74 (51.4%)	803 (48.3%)	

Based on Pearson’s chi-square test, statistical significance (*p* < 0.05) is shown in bold. Abbreviation: DFS: disease-free survival, BCSS: breast cancer-specific survival, BCS: breast-conserving surgery, MRM: modified radical mastectomy, ER: estrogen receptor, PgR: progesterone receptor.

**Table 2:** Cox analysis for BCSS and DFS analysis within 5 years.

Characteristic		BCSS				DFS			
	*n*	Univariate		Multivariate		Univariate		Multivariate	
	1807	HR (95% CI) ^a^	*p*	HR (95% CI) ^a^	*p*	HR (95% CI) ^a^	*p*	HR (95% CI) ^a^	*p*
**Age**									
<50	755	1 (ref)	-			1 (ref)	-		
≥50	1052	1.12 (0.689–1.821)	0.647			1.125 (0.806–1.571)	0.489		
**Operation type**									
BCS	685	1 (ref)	-	1 (ref)	-	1 (ref)	-	1 (ref)	-
MRM	1122	**3.261 (1.71–6.218)**	**<0.001**	1.849 (0.951–3.595)	0.07	**2.413 (1.614–3.608)**	**<0.001**	**1.596 (1.054–2.418)**	**0.027**
**Tumor size (cm)**			**<0.001**		**<0.001**		**<0.001**		**<0.001**
≤2	1186	1 (ref)	-	1 (ref)	-	1 (ref)	-	1 (ref)	-
>2, ≤5	552	**6.104 (3.314–11.241)**	**<0.001**	**3.688 (1.871–7.267)**	**<0.001**	**3.817 (2.642–5.514)**	**<0.001**	**2.569 (1.682–3.922)**	**<0.001**
>5	69	**19.335 (9.333–40.056)**	**<0.001**	**9.204 (4.05–20.919)**	**<0.001**	**9.552 (5.736–15.907)**	**<0.001**	**5.408 (3.028–9.661)**	**<0.001**
**Lymph node**									
Negative	1293	1 (ref)	-	1 (ref)	-	1 (ref)	-	1 (ref)	-
Positive	514	**5.704 (3.41–9.541)**	**<0.001**	**2.814 (1.535–5.161)**	**<0.001**	**3.455 (2.484–4.805)**	**<0.001**	**1.617 (1.089–2.403)**	**0.017**
**Histological subtype**			0.177		0.709		**0.031**		0.347
Ductal	1484	1 (ref)	-			1 (ref)	-	1 (ref)	-
Lobular	114	1.251 (0.500–3.133)	0.632			1.132 (0.593–2.16)	0.708	0.934 (0.472–1.849)	0.845
Mixed (ductal and lobular)	139	1.877 (0.925–3.809)	0.081			**1.703 (1.036–2.8)**	**0.036**	1.336 (0.802–2.226)	0.265
Others	70	0.805 (0.196–3.304)	0.763			0.365 (0.09–1.477)	0.158	0.376 (0.092–1.546)	0.175
**Histologic grade**			**0.005**		0.347		**0.002**		0.706
1	273	1 (ref)	-	1 (ref)	-	1 (ref)	-	1 (ref)	-
2	640	2.605 (0.767–8.845)	0.125	1.315 (0.38–4.55)	0.666	**2.563 (1.211–5.424)**	**0.014**	1.362 (0.629–2.951)	0.433
3	889	**4.787 (1.49–15.38)**	**0.009**	1.86 (0.557–6.208)	0.313	**3.481 (1.689–7.177)**	**<0.001**	1.377 (0.643–2.95)	0.41
Unknown	5					-		-	
**ER**									
Negative	267	1 (ref)	-	1 (ref)	-	1 (ref)	-	1 (ref)	-
≥1%	1540	**0.56 (0.32–0.98)**	**0.042**	**0.482 (0.26–0.895)**	**0.021**	**0.594 (0.401–0.88)**	**0.009**	**0.513 (0.331–0.794)**	**0.003**
**PgR**									
Negative	165	1 (ref)	-	1 (ref)	-	1 (ref)	-	1 (ref)	-
≥1%	1642	**0.347 (0.196–0.616)**	**<0.001**	**0.298 (0.162–0.548)**	**<0.001**	**0.472 (0.304–0.731)**	**<0.001**	**0.389 (0.246–0.615)**	**<0.001**
**Hormone therapy type**			**<0.001**		0.706		**<0.001**		**<0.001**
Anti-estrogens	1146	1 (ref)	-	1 (ref)	-	1 (ref)	-	1 (ref)	-
Enzyme-inhibitors	195	**3.475 (1.88–6.424)**	**<0.001**	1.298 (0.676–2.49)	0.433	**3.694 (2.313–5.898)**	**<0.001**	**1.981 (1.202–3.265)**	**0.007**
Both	466	**2.068 (1.199–3.567)**	**0.009**	1.032 (0.581–1.833)	0.915	**3.727 (2.573–5.4)**	**<0.001**	**2.417 (1.627–3.593)**	**<0.001**
**Chemotherapy**									
No	792	1 (ref)	-	1 (ref)	-	1 (ref)	-	1 (ref)	-
Yes	1015	**3.574 (1.916–6.667)**	**<0.001**	0.829 (0.405–1.696)	0.607	**2.632 (1.785–3.882)**	**<0.001**	0.811 (0.506–1.298)	0.382
**Radiotherapy**									
No	930	1 (ref)	-			1 (ref)	-		
Yes	877	1.57 (0.966–2.551)	0.069			1.097 (0.791–1.521)	0.58		

^a^ Based on the Cox regression model, statistical significance (*p* < 0.05) is shown in bold. Abbreviation: DFS: disease-free survival, BCSS: breast cancer-specific survival, HR: hazard ratio, CI: confidence interval, BCS: breast-conserving surgery, MRM: modified radical mastectomy, ER: estrogen receptor, PgR: progesterone receptor.

**Table 3:** Cox analysis for BCSS and DFS analysis within 10 years.

Characteristic		BCSS				DFS			
	*n*	Univariate		Multivariate		Univariate		Multivariate	
	717	HR (95% CI) ^a^	*p*	HR (95% CI) ^a^	*p*	HR (95% CI) ^a^	*p*	HR (95% CI) ^a^	*p*
**Age**									
<50	333	1 (ref)	-			1 (ref)	-		
≥50	384	1.137 (0.697–1.855)	0.606			0.97 (0.646–1.457)	0.883		
**Operation type**									
BCS	284	1 (ref)	-	1 (ref)	-	1 (ref)	-	1 (ref)	-
MRM	433	**3.173 (1.695–5.939)**	**<0.001**	**1.952 (1.187–3.209)**	**0.008**	**2.333 (1.446–3.764)**	**<0.001**	**1.484 (1.047–2.104)**	**0.027**
**Tumor size (cm)**			**<0.001**		**<0.001**		**<0.001**		**<0.001**
≤2	448	1 (ref)	-	1 (ref)	-	1 (ref)	-	1 (ref)	-
>2, ≤5	239	**4.663 (2.6–8.363)**	**<0.001**	**2.654 (1.64–4.295)**	**<0.001**	**3.654 (2.32–5.755)**	**<0.001**	**2.367 (1.655–3.384)**	**<0.001**
>5	30	**11.627 (5.394–25.065)**	**<0.001**	**5.821 (3.128–10.831)**	**<0.001**	**7.468 (3.81–14.639)**	**<0.001**	**4.533 (2.712–7.576)**	**<0.001**
**Lymph node**									
Negative	512	1 (ref)	-	1 (ref)	-	1 (ref)	-	1 (ref)	-
Positive	205	**5.155 (3.084–8.617)**	**<0.001**	**2.496 (1.564–3.984)**	**<0.001**	**3.393 (2.253–5.11)**	**<0.001**	**1.578 (1.117–2.23)**	**0.01**
**Histological subtype**			**0.01** **9**		0.493		0.081		0.256
Ductal	626	1 (ref)	-	1 (ref)	-	1 (ref)	-	1 (ref)	-
Lobular	35	**2.576 (1.168–5.681)**	**0.019**	1.023 (0.494–2.12)	0.951	**2.264 (1.134–4.521)**	**0.021**	0.913 (0.513–1.625)	0.757
Mixed (ductal and lobular)	30	**2.732 (1.171–6.372)**	**0.02**	1.501 (0.875–2.575)	0.14	1.764 (0.768–4.052)	0.181	1.423 (0.932–2.173)	0.102
Others	26	0.961 (0.234–3.951)	0.957	0.803 (0.247–2.614)	0.716	0.96 (0.303–3.044)	0.945	0.604 (0.22–1.662)	0.329
**Histologic grade**			0.111		0.314		**0.02** **4**		0.226
1	113	1 (ref)	-	1 (ref)	-	1 (ref)	-	1 (ref)	-
2	244	**2.997 (1.043–8.612)**	**0.042**	1.881 (0.654–5.408)	0.241	**3.268 (1.386–7.709)**	**0.007**	1.872 (0.918–3.82)	0.085
3	357	**2.918 (1.039–8.199)**	**0.042**	2.168 (0.765–6.142)	0.145	**2.584 (1.105–6.045)**	**0.029**	1.749 (0.863–3.545)	0.121
Unknown	3					-		-	
**ER**									
Negative	135	1 (ref)	-			1 (ref)	-		
≥1%	582	0.785 (0.441–1.399)	0.412			0.794 (0.488–1.291)	0.352		
**PgR**									
Negative	82	1 (ref)	-	1 (ref)	-	1 (ref)	-	1 (ref)	-
≥1%	635	**0.484 (0.263–0.888)**	**0.019**	**0.374 (0.234–0.597)**	**<0.001**	**0.572 (0.334–0.98)**	**0.042**	**0.466 (0.314–0.693)**	**<0.001**
**Hormone therapy type**			**<0.001**		**0.018**		**<0.001**		**<0.001**
Anti-estrogens	497	1 (ref)	-	1 (ref)	-	1 (ref)	-	1 (ref)	-
Enzyme-inhibitors	44	**2.862 (1.246–6.571)**	**0.013**	1.707 (0.987–2.954)	0.056	**3.663 (1.865–7.196)**	**<0.001**	**2.227 (1.43–3.467)**	**<0.001**
Both	176	**3.12 (1.862–5.227)**	**<0.001**	**1.892 (1.21–2.956)**	**0.005**	**3.707 (2.397–5.734)**	**<0.001**	**2.686 (1.895–3.807)**	**<0.001**
**Chemotherapy**									
No	294	1 (ref)	-	1 (ref)	-	1 (ref)	-	1 (ref)	-
Yes	423	**2.694 (1.467–4.947)**	**0.001**	0.985 (0.564–1.72)	0.957	**2.248 (1.393–3.626)**	**<0.001**	0.904 (0.602–1.358)	0.628
**Radiotherapy**									
No	357	1 (ref)	-			1 (ref)	-		
Yes	360	1.02 (0.627–1.659)	0.937			1.106 (0.736–1.663)	0.627		

^a^ Based on the Cox regression model, statistical significance (*p* < 0.05) is shown in bold. Abbreviation: DFS: disease-free survival, BCSS: breast cancer-specific survival, HR: hazard ratio, CI: confidence interval, BCS: breast-conserving surgery, MRM: modified radical mastectomy, ER: estrogen receptor, PgR: progesterone receptor.

## Data Availability

Due to legal restrictions imposed by the government of Taiwan in relation to the “Personal Information Protection Act”, data cannot be made publicly available. Requests for data can be sent as a formal proposal to the the Cancer Registry of Tri-Service General Hospital.

## Supplementary Materials

The following are available online at https://www.mdpi.com/2072-6694/13/4/905/s1, Figure S1: Distribution of hazard ratio based on the ER status within 10 years of follow-up. The hazard ratios were calculated after adjusting significant covariates under univariate analysis in Table 3. A total of 135 patients with negative ER expression but positive PgR expression were defined as the reference group; 15 patients died from BCSS events and 21 exhibited DFS events during the period in the reference group. Hazard ratios for BCSS events by subgroups of ER percentage (A) and Allred score (B). Hazard ratios for DFS events by subgroups of ER percentage (C) and Allred score (D). Figure S2: Immunohistochemical staining results of breast cancer patients in our hospital (200× magnification). (A) ER positive for 20% tumor cells, intensity 2+. (B) ER positive for 90% tumor cells, intensity 3+. (C) PgR positive for 20% tumor cells, intensity 1+. (B) PgR positive for 90% tumor cells, intensity 3+. Abbreviation: ER: estrogen receptor, PgR: progesterone receptor.
