# Supplementary material for: Amongst Women Stratified to Receive Endocrine Therapy on the Basis of Their Tumor Estrogen and Progesterone Receptor Levels, Those with Higher Tumor Progesterone Receptor Levels Had a Better Outcome Than Those with Lower Levels of Tumor Progesterone Receptor
Source: Cancers (Basel). 2021 Feb 21;13(4):905. doi: 10.3390/cancers13040905 (PMC7926358; doi:10.3390/cancers13040905)
Supplement: Supplementary file 1 [file cancers-13-00905-s001.zip › cancers-1053297-supplementary/Supplementary Figure S2.pdf]

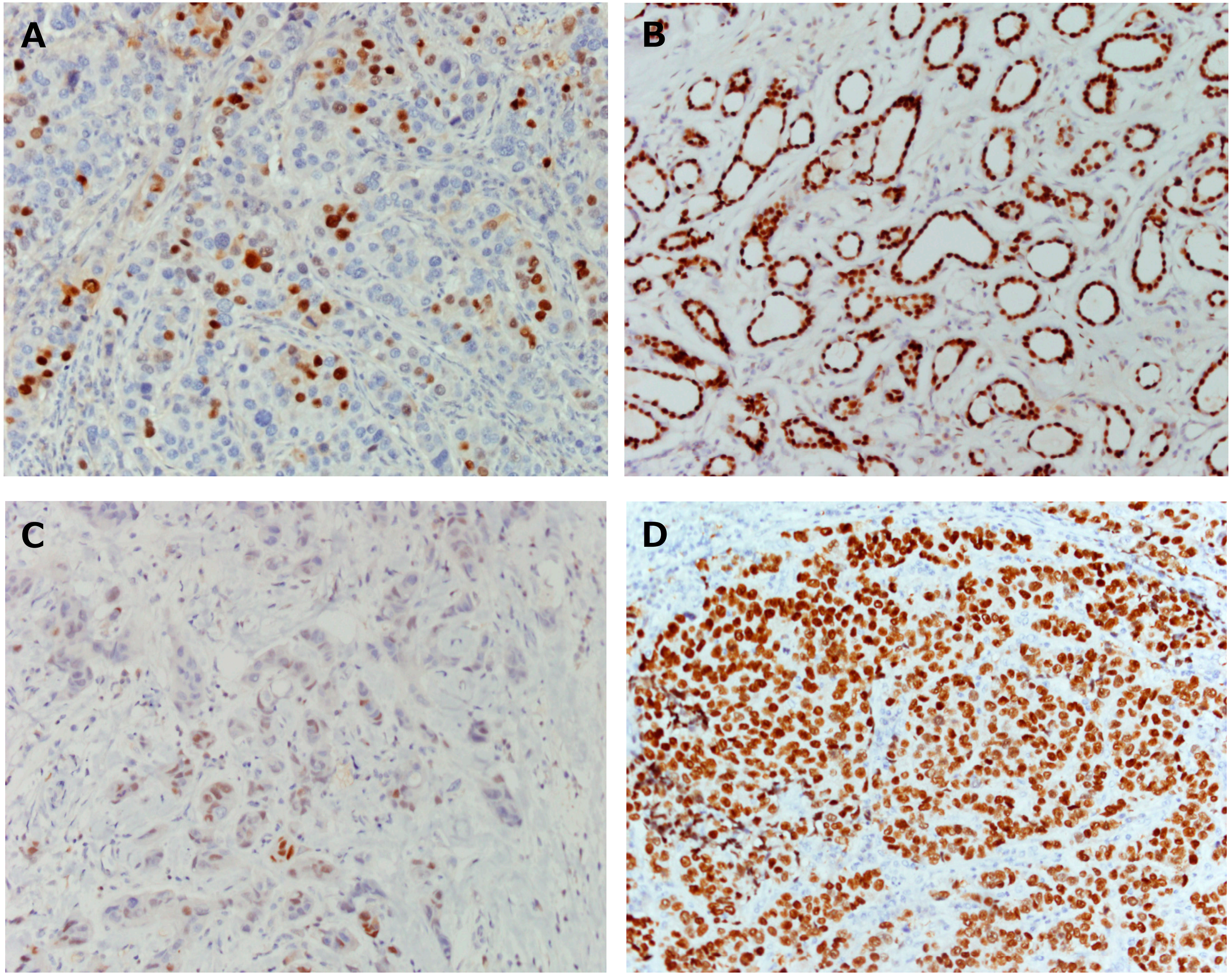

Figure S2. Immunohistochemical staining results of breast cancer patients in our hospital (200× magnification).

(A)ER positive for 20% tumor cells, intensity 2+. (B) ER positive for 90% tumor cells, intensity 3+.

(C)PgR positive for 20% tumor cells, intensity 1+. (B) PgR positive for 90% tumor cells, intensity 3+.

Abbreviation: ER: estrogen receptor, PgR: progesterone receptor.
