# Supplementary material for: Amongst Women Stratified to Receive Endocrine Therapy on the Basis of Their Tumor Estrogen and Progesterone Receptor Levels, Those with Higher Tumor Progesterone Receptor Levels Had a Better Outcome Than Those with Lower Levels of Tumor Progesterone Receptor
Source: Cancers (Basel). 2021 Feb 21;13(4):905. doi: 10.3390/cancers13040905 (PMC7926358; doi:10.3390/cancers13040905)
Supplement: Supplementary file 1 [file Supplementary Figure S1.pdf › cancers-1053297-supplementary/Supplementary Figure S1.pdf]

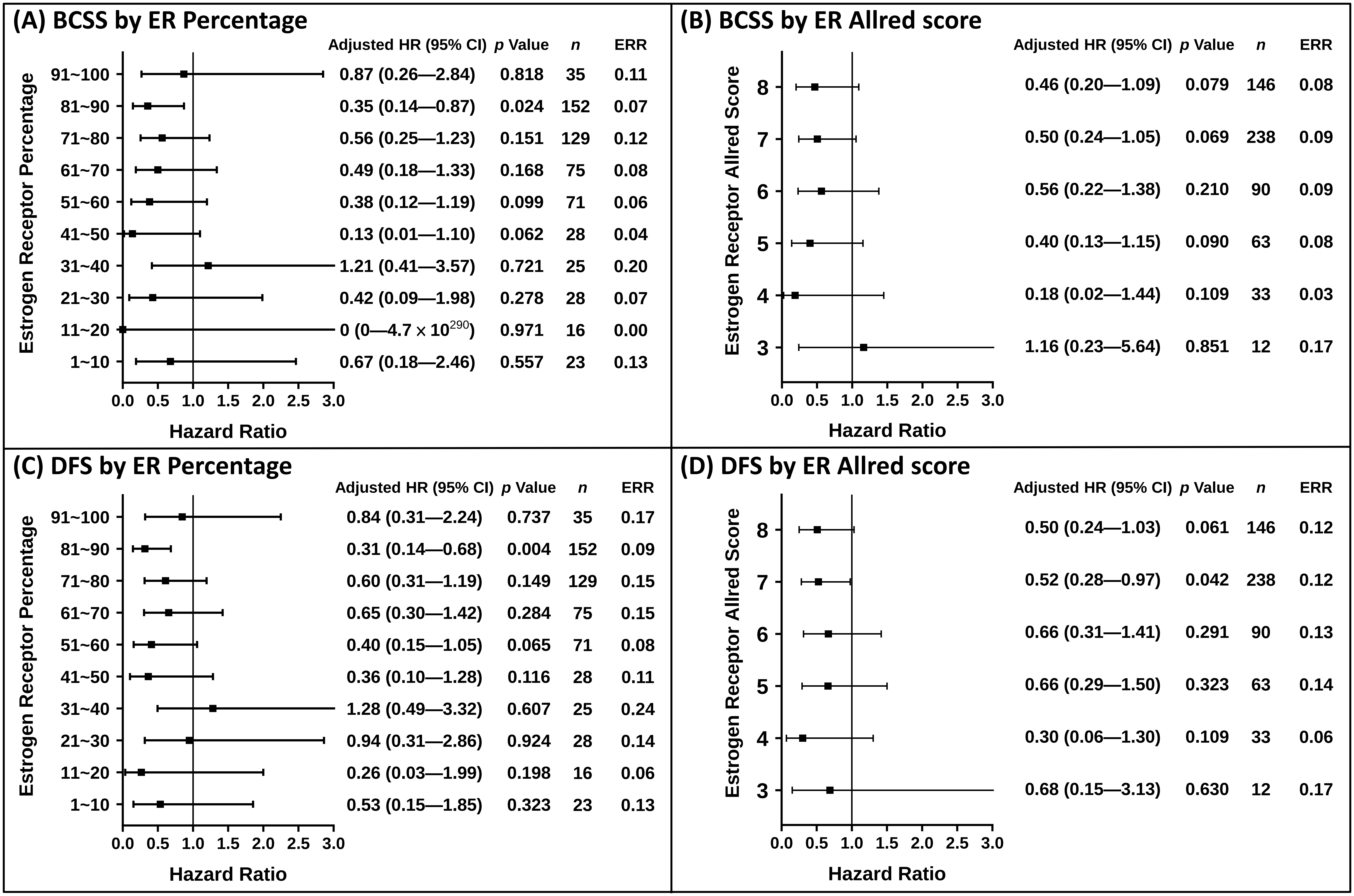

Supplementary Figure S1. Distribution of hazard ratio based on the ER status within 10 years of follow-up. The hazard ratios were calculated after adjusting significant covariates under univariate analysis in Table 3. A total of 135 patients with negative ER expression but positive PgR expression were defined as the reference group; 15 patients died from BCSS events and 21 exhibited DFS events during the period in the reference group. Hazard ratios for BCSS events by subgroups of ER percentage (A) and Allred score (B). Hazard ratios for DFS events by subgroups of ER percentage (C) and Allred score (D).
